# A dose-dependent perturbation in cardiac energy metabolism is linked to radiation-induced ischemic heart disease in Mayak nuclear workers

**DOI:** 10.18632/oncotarget.10424

**Published:** 2016-07-06

**Authors:** Omid Azimzadeh, Tamara Azizova, Juliane Merl-Pham, Vikram Subramanian, Mayur V. Bakshi, Maria Moseeva, Olga Zubkova, Stefanie M. Hauck, Nataša Anastasov, Michael J. Atkinson, Soile Tapio

**Affiliations:** ^1^ Helmholtz Zentrum München-German Research Center for Environmental Health (GmbH), Institute of Radiation Biology, Neuherberg, Germany; ^2^ Southern Urals Biophysics Institute, Russian Federation, Ozyorsk, Russia; ^3^ Helmholtz Zentrum München-German Research Center for Environmental Health (GmbH), Research Unit Protein Science, Munich, Germany; ^4^ Chair of Radiation Biology, Technical University of Munich, Munich, Germany

**Keywords:** ionising radiation, proteomics, heart disease, PPAR alpha, mitochondrial dysfunction

## Abstract

Epidemiological studies show a significant increase in ischemic heart disease (IHD) incidence associated with total external gamma-ray dose among Mayak plutonium enrichment plant workers. Our previous studies using mouse models suggest that persistent alteration of heart metabolism due to the inhibition of peroxisome proliferator-activated receptor (PPAR) alpha accompanies cardiac damage after high doses of ionising radiation. The aim of the present study was to elucidate the mechanism of radiation-induced IHD in humans. The cardiac proteome response to irradiation was analysed in Mayak workers who were exposed only to external doses of gamma rays. All participants were diagnosed during their lifetime with IHD that also was the cause of death. Label-free quantitative proteomics analysis was performed on tissue samples from the cardiac left ventricles of individuals stratified into four radiation dose groups (0 Gy, < 100 mGy, 100–500 mGy, and > 500 mGy). The groups could be separated using principal component analysis based on all proteomics features. Proteome profiling showed a dose-dependent increase in the number of downregulated mitochondrial and structural proteins. Both proteomics and immunoblotting showed decreased expression of several oxidative stress responsive proteins in the irradiated hearts. The phosphorylation of transcription factor PPAR alpha was increased in a dose-dependent manner, which is indicative of a reduction in transcriptional activity with increased radiation dose. These data suggest that chronic external radiation enhances the risk for IHD by inhibiting PPAR alpha and altering the expression of mitochondrial, structural, and antioxidant components of the heart.

## INTRODUCTION

Mayak Production Association (PA), located 150 km south-east of Ekaterinburg, is one of the biggest nuclear facilities in the Russian Federation. Individual dosimetric monitoring of external exposure performed at Mayak PA showed that the total external gamma-ray doses ranged widely from below 100 mGy to more than 5 Gy, with 32.6% of the workers having a total dose greater than 1 Gy [1]. Epidemiological studies in this cohort showed a significant increase in ischemic heart disease (IHD) incidence associated with total external gamma-ray dose after correction for multiple competing factors such as smoking and alcohol consumption [1–3]. The risk estimates for IHD in relation to chronic external radiation dose are generally compatible with those reported in other large occupational studies and the Japanese A-bomb survivors [4].

Mitochondrial dysfunction plays a key role in the pathogenesis of IHD [5]. A high rate of mitochondrial catabolism of carbohydrates and fatty acids is crucial for furnishing the energy supply necessary for heart function [6]. Under normal conditions the adult heart relies mostly on fatty acids for this energy production via the oxidative phosphorylation (OXPHOS) process, with only 10% to 30% of total ATP being derived from glucose [7]. However, a normal heart can easily switch between fatty acids and glucose for ATP production, depending on energy demand and substrate availability [8]. In pathological conditions such as IHD this flexibility is lost and either superseded by a preference for glucose over fat [9] or an overall reduction of mitochondrial oxidative metabolism independent of the energy source [10]. Both scenarios are associated with the reduction in the level of active peroxisome proliferator-activated receptor (PPAR) alpha in cardiac ventricles [11]. PPAR alpha functions as a key regulator of cardiac metabolism and is essential for fatty acid oxidation [6].

We have previously shown that local heart irradiation in mice persistently decreases the respiratory capacity of cardiac mitochondria [12, 13], reduces their number, and results in damage to the cristae structure [14]. Importantly, the activity of the transcription factor PPAR alpha is reduced by a dose-dependent increase in phosphorylation [14].

Although mouse models are widely used to study cardiac disease, there are functional differences between mouse and human hearts [15]. Infarction is virtually unknown in mice, probably due to their short life span, and differences in the heart physiology and diet. Even though mouse models have led to important observations on the causes of radiation-induced IHD, the question of their clinical relevance remains.

The aim of the present study was to examine whether alteration in cardiac metabolism, and its key regulator PPAR alpha, contribute to radiation-induced IHD in man. Here, we investigated human left ventricle proteome profiles in Mayak workers who had been occupationally exposed to different cumulative doses of external gamma rays. All participants had previously been diagnosed with IHD that also was the primary cause of death [1, 16]. The proteomic analysis revealed a dose-dependent series of alteration in the levels of proteins involved in the left ventricle function and structure. These include proteins critical for mitochondrial energy metabolism and cardiac cytoskeleton. A significant inactivation of PPAR alpha by phosphorylation was observed in the highest dose group (> 500 mGy). The present study provides, for the first time, a proteomics signature of radiation-induced human heart ischemia. This is coherent with the observations made using irradiated mice upon the radiation dose.

## RESULTS

### Chronic irradiation alters the heart proteome in a dose-dependent manner

Global proteomics analysis identified 1,281 proteins in total (Supplementary Table S1). Of the quantified proteins, 101, 225 and 431 proteins were significantly changed in expression (2 unique peptides; fold change > 1.30 or < 0.77; q < 0.05) after exposure to doses of < 100 mGy, 100–500 mGy and > 500 mGy, respectively. This indicated a dose-dependent increase in the number of deregulated proteins (Supplementary Tables S2–S4), as seen in irradiated mouse heart models [14]. A large number (72) of deregulated proteins were shared between all three irradiated groups compared to the control (Table 1). The majority of these shared proteins belonged to mitochondria (24 proteins) or cytoskeleton (13 proteins).

**Table 1:** Significantly deregulated proteins shared in all radiation dose groups

#	Symbol	Entrez Gene Name	ratio	ratio	ratio	GO - Molecular function
			< 100 mGy/ control	100–500 mGy/ controls	> 500 mGy/ controls	
1	ACAT1	acetyl-CoA acetyltransferase 1	0.77	0.68	0.42	metabolic activity (GO:0003824)
2	AHSG	alpha-2-HS-glycoprotein	0.47	0.63	0.33	metabolic activity (GO:0003824)
3	AIFM1	apoptosis-inducing factor, mitochondrion-associated, 1	0.65	0.62	0.40	antioxidant activity (GO:0016209)
4	AK2	adenylate kinase 2	0.74	0.76	0.48	metabolic activity (GO:0003824)
5	ALDOA	aldolase A, fructose-bisphosphate	0.63	0.63	0.42	metabolic activity (GO:0003824)
6	ANXA11	annexin A11	0.77	0.73	0.53	structural molecule activity (GO:0005198)
7	ATP5B	ATP synthase, H+ transporting, beta	0.62	0.62	0.38	metabolic activity (GO:0003824)
8	CCT3	chaperonin containing TCP1, subunit 3 (gamma)	0.65	0.76	0.52	ATP binding (GO:0005524)
9	CHCHD3	coiled-coil-helix-coiled-coil-helix domain containing 3	0.46	0.6	0.34	structural molecule activity (GO:0005198)
10	CKM	creatine kinase, muscle	0.66	0.65	0.38	metabolic activity (GO:0003824)
11	COQ9	coenzyme Q9	0.73	0.55	0.38	metabolic activity (GO:0003824)
12	DBI	GABA receptor modulator, acyl-CoA binding protein	0.73	0.68	0.41	metabolic activity (GO:0003824)
13	DECR1	2,4-dienoyl CoA reductase 1, mitochondrial	0.7	0.57	0.39	metabolic activity (GO:0003824)
14	DLD	dihydrolipoamide dehydrogenase	0.74	0.64	0.51	metabolic activity (GO:0003824)
15	ECI1	enoyl-CoA delta isomerase 1	0.7	0.65	0.44	metabolic activity (GO:0003824)
16	FH	fumarate hydratase	0.71	0.61	0.40	metabolic activity (GO:0003824)
17	GLRX5	glutaredoxin 5	0.52	0.5	0.37	antioxidant activity (GO:0016209)
18	HADH	hydroxyacyl-CoA dehydrogenase	0.67	0.58	0.37	metabolic activity (GO:0003824)
19	HNRNPA1L2	heterogeneous nuclear ribonucleoprotein A1-like 2	0.77	0.73	0.56	nucleotide binding (GO:0000166)
20	HNRNPA2B1	heterogeneous nuclear ribonucleoprotein A2/B1	0.77	0.77	0.44	nucleotide binding (GO:0000166)
21	HSD17B10	hydroxysteroid (17-beta) dehydrogenase 10	0.76	0.66	0.42	metabolic activity (GO:0003824)
22	LDHB	lactate dehydrogenase B	0.78	0.6	0.49	metabolic activity (GO:0003824)
23	LGALS1	lectin, galactoside-binding, soluble, 1	0.59	0.53	0.32	nucleotide binding (GO:0000166)
24	LGALS3	lectin, galactoside-binding, soluble, 3	0.48	0.49	0.29	nucleotide binding (GO:0000166)
25	MCCC1	methylcrotonoyl-CoA carboxylase 1 (alpha)	0.62	0.52	0.39	ATP binding (GO:0005524)
26	MCEE	methylmalonyl CoA epimerase	0.64	0.63	0.47	metabolic activity (GO:0003824)
27	MDH2	malate dehydrogenase 2, NAD (mitochondrial)	0.73	0.68	0.44	metabolic activity (GO:0003824)
28	ME3	malic enzyme 3, NADP(+)-dependent, mitochondrial	3.8	6.21	4.45	metabolic activity (GO:0003824)
29	MECR	mitochondrial trans-2-enoyl-CoA reductase	0.62	0.55	0.38	metabolic activity (GO:0003824)
30	MYBPC3	myosin binding protein C, cardiac	0.74	0.57	0.45	structural molecule activity (GO:0005198)
31	MYH10	myosin, heavy chain 10, non-muscle	2.16	2.91	1.92	structural molecule activity (GO:0005198)
32	MYH11	myosin, heavy chain 11, smooth muscle	0.45	0.56	0.52	structural molecule activity (GO:0005198)
33	MYL2	myosin, light chain 2, regulatory, cardiac, slow	0.63	0.65	0.40	structural molecule activity (GO:0005198)
34	MYL3	myosin, light chain 3, alkali; ventricular, skeletal, slow	0.67	0.65	0.41	structural molecule activity (GO:0005198)
35	MYL6	myosin light chain 6,smooth muscle and non-muscle	0.54	0.68	0.38	structural molecule activity (GO:0005198)
36	MYOM1	myomesin 1	0.76	0.58	0.44	structural molecule activity (GO:0005198)
37	MYOM2	myomesin 2	0.7	0.51	0.39	structural molecule activity (GO:0005198)
38	NDUFA3	NADH dehydrogenase (ubiquinone) 1 alpha	0.41	0.56	0.29	metabolic activity (GO:0003824)
39	NID1	nidogen 1	0.69	0.67	0.42	extracellular matrix binding (GO:0050840)
40	NMT1	N-myristoyltransferase 1	3.4	16.63	23.75	apoptotic activity (GO:0006915)
41	NPM1	nucleolar phosphoprotein B23, numatrin	0.72	0.77	0.44	histone binding (GO:0042393)
42	PARK7	protein deglycase DJ-1	0.73	0.72	0.46	nucleic acid binding transcription factor activity (GO:0001071)
43	PCMT1	protein-L-isoaspartate (D-aspartate) O-methyltransferase	0.71	0.68	0.53	metabolic activity (GO:0003824)
44	PDHA1	pyruvate dehydrogenase (lipoamide) alpha 1	0.77	0.63	0.47	metabolic activity (GO:0003824)
45	PDHB	pyruvate dehydrogenase (lipoamide) beta	0.76	0.56	0.46	metabolic activity (GO:0003824)
46	PGAM2	phosphoglycerate mutase 2 (muscle)	0.7	0.55	0.36	metabolic activity (GO:0003824)
47	PGK1	phosphoglycerate kinase 1	0.67	0.59	0.40	metabolic activity (GO:0003824)
48	PGM1	phosphoglucomutase 1	0.7	0.48	0.35	metabolic activity (GO:0003824)
49	PKM	pyruvate kinase, muscle	0.73	0.56	0.38	metabolic activity (GO:0003824)
50	PLEC	plectin	0.73	0.64	0.43	structural molecule activity (GO:0005198)
51	PPP1CB	protein phosphatase 1, beta isozyme	1.61	2.09	1.62	structural molecule activity (GO:0005198)
52	PRDX3	peroxiredoxin 3	0.76	0.73	0.49	antioxidant activity (GO:0016209)
53	PRDX5	peroxiredoxin 5	0.74	0.64	0.40	antioxidant activity (GO:0016209)
54	PRDX6	peroxiredoxin 6	0.74	0.72	0.47	antioxidant activity (GO:0016209)
55	PRKAR1A	protein kinase, cAMP-dependent, regulatory, type I, A	0.73	0.65	0.50	cAMP binding (GO:0030552)
56	PSMA5	proteasome (prosome, macropain) subunit, alpha type, 5	0.71	0.67	0.41	protein polyubiquitination (GO:0000209)
57	PSMA6	proteasome (prosome, macropain) subunit, alpha type, 6	0.64	0.62	0.46	protein polyubiquitination (GO:0000209)
58	PTRF	polymerase I and transcript release factor	0.65	0.65	0.46	poly(A) RNA binding (GO:0044822)
59	PYGM	phosphorylase, glycogen, muscle	0.54	0.49	0.24	metabolic activity (GO:0003824)
60	RPS27A	ribosomal protein S27a	0.74	0.62	0.44	structural molecule activity (GO:0005198)
61	SDPR	serum deprivation response	0.71	0.75	0.48	protein kinase C binding (GO:0005080)
62	SOD2	superoxide dismutase 2, mitochondrial	0.73	0.61	0.42	antioxidant activity (GO:0016209)
63	SPTA1	spectrin, alpha, erythrocytic 1	4.36	4.62	4.87	structural molecule activity (GO:0005198)
64	SUCLG1	succinate-CoA ligase, alpha subunit	0.72	0.74	0.45	metabolic activity (GO:0003824)
65	SUCLG2	succinate-CoA ligase, beta subunit	0.66	0.67	0.42	metabolic activity (GO:0003824)
66	TOM1L2	target of myb1 like 2 membrane trafficking protein	1.8	1.83	1.50	metabolic activity (GO:0003824)
67	TPM2	tropomyosin 2 (beta)	0.49	0.5	0.33	structural molecule activity (GO:0005198)
68	TUBA8	tubulin, alpha 8	0.55	0.56	0.38	structural molecule activity (GO:0005198)
69	TXN	thioredoxin	0.69	0.54	0.38	antioxidant activity (GO:0016209)
70	UQCR10	ubiquinol-cytochrome c reductase, subunit X	0.56	0.46	0.37	metabolic activity (GO:0003824)
71	UQCRC2	ubiquinol-cytochrome c reductase core protein II	0.63	0.57	0.39	metabolic activity (GO:0003824)
72	VCAN	versican	3.05	4.11	2.31	structural molecule activity (GO:0005198)

The accession number, protein ID, full name and fold change after exposure to < 100 mGy, 100–500 mGy or > 500 mGy is shown for each protein.

**Figure 1:**
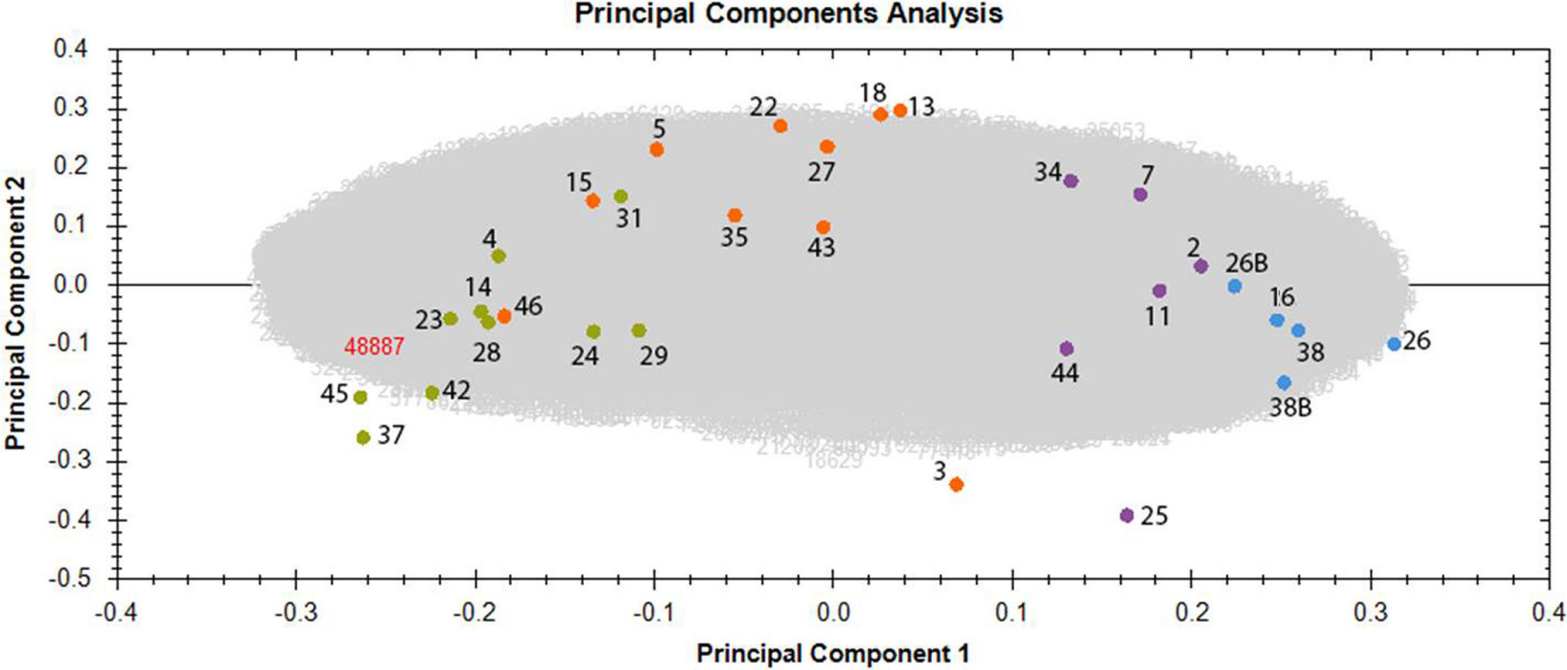
Principal component analysis (PCA) based on all proteomic features in the left ventricle of sample donors in different dose groups The PCA used features with charges +2 to +7 resulting in PC1 and PC2 as follows: PC1 23.65% and PC2 8.36%. The control samples with the corresponding donor number are represented as blue spots, the samples exposed to < 100 mGy in purple, the samples exposed to 100–500 mGy in orange and the samples exposed to > 500 mGy in green. Samples number 26 and 38 were run as 2 technical replicates and are indicated as 26, 26B and 38, 38B. Detailed information of the sample donors and the exact doses are given in Supplementary Table S10. The analysis was performed using the Progenesis QI software (http://www.nonlinear.com).

To investigate differences in the proteome profiles between the different dose groups, a PCA based on all proteomics features was performed. Control and irradiated samples clustered into four groups according to the dose (Figure 1). The distance between the cluster that represents the control group and the clusters representing the irradiated groups increased with increasing dose. Even though the workers exposed to the highest dose (> 500 mGy) were generally older than the members of other groups, the PCA did not show any clustering based on age. Similarly, no clustering was observed based on smoking status or index, alcohol consumption, or body mass index (Supplementary Table S10).

Some outliers were identified in each irradiated group, namely donors 3, 25 and 46 (Figure 1). Sample number 25, belonging to the group < 100 mGy, was exposed to the very low dose of 6 mGy, and unsurprisingly showed proteomics features that were more similar to those of the control group. Sample number 3, belonging to the dose group of 100–500 mGy, was exposed to the dose of 114 mGy and showed similarity with the group of < 100 mGy. Sample number 46, a member of the dose group 100–500 mGy, was exposed to the dose of 483 mGy, and was placed in close proximity to the group exposed to the highest dose (> 500 mGy) (Figure 1 and Supplementary Table S10). These deviations strengthen the evidence for a dose-response relationship.

**Figure 2:**
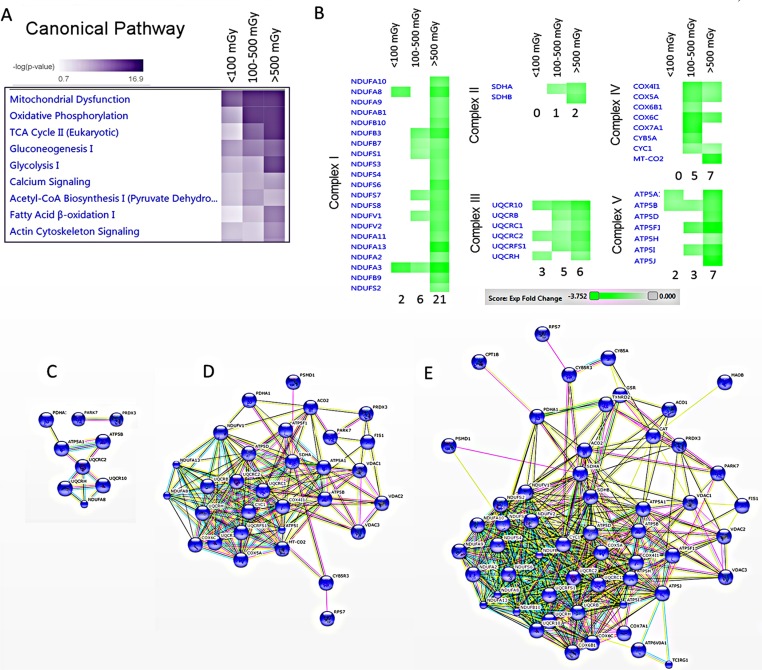
Pathway and network analysis of significantly differentially expressed mitochondrial proteins A dose-dependent alteration is observed in the pathways involved in the energy production. The pathway scores are displayed using a purple colour gradient, where darker purple corresponds to higher scores (increased statistical significance). The score is the negative log of the *p*-value derived from the Fisher′s Exact test. By default, the rows (pathways) with the highest total score across the set of observations are sorted to the top (**A**). Heat map for the expression values of differentially expressed OXPHOS proteins between dose groups is displayed using a green colour gradient for downregulated proteins, where dark green corresponds to large downregulation. The numbers shows how many proteins were deregulated in each subunit (**B**) (http://www.INGENUITY.com). Protein-protein interaction analysis of the significantly differentially expressed proteins showing the networks of deregulated mitochondrial proteins in the dose groups < 100 mGy (**C**), 100–500 mGy (**D**) and > 500 mGy (**E**) (http://string-db.org).

A detailed analysis of functional interactions and biological pathways was performed using IPA (http://www.INGENUITY.com) (Supplementary Tables S5 and S6). Mitochondrial dysfunction and metabolic impairment were indicated in all irradiated groups compared to the control group (Figure 2A). A dose-dependent reduction was found in the expression of proteins of the respiratory complexes I, III and V. The complexes II and IV were affected only in the two high-dose groups (Figure 2B). The number of deregulated mitochondrial proteins increased with the radiation dose (Figure 2C–2E and Supplementary Table S6).

Several proteins belonging to energy production pathways associated with fatty acid oxidation (lipid metabolism, Krebs cycle) were downregulated by irradiation (Figure 2A and Supplementary Table S6). Also several enzymes in the glycolysis pathway were downregulated (Supplementary Figure S1 and Supplementary Table S6), suggesting a general depletion of energy supply, rather than a glucose/lipid switch.

In addition, the number of deregulated proteins belonging to actin cytoskeleton or calcium signalling was increased in a dose-dependent manner (Supplementary Figure S2 and Supplementary Table S6). The majority of significantly altered proteins were associated with heart diseases including left ventricle dysfunction and heart hypertrophy (Supplementary Figure S3 and Supplementary Table S7).

### Immunoblotting confirms radiation-induced downregulation of structural and antioxidant proteins

**Figure 3:**
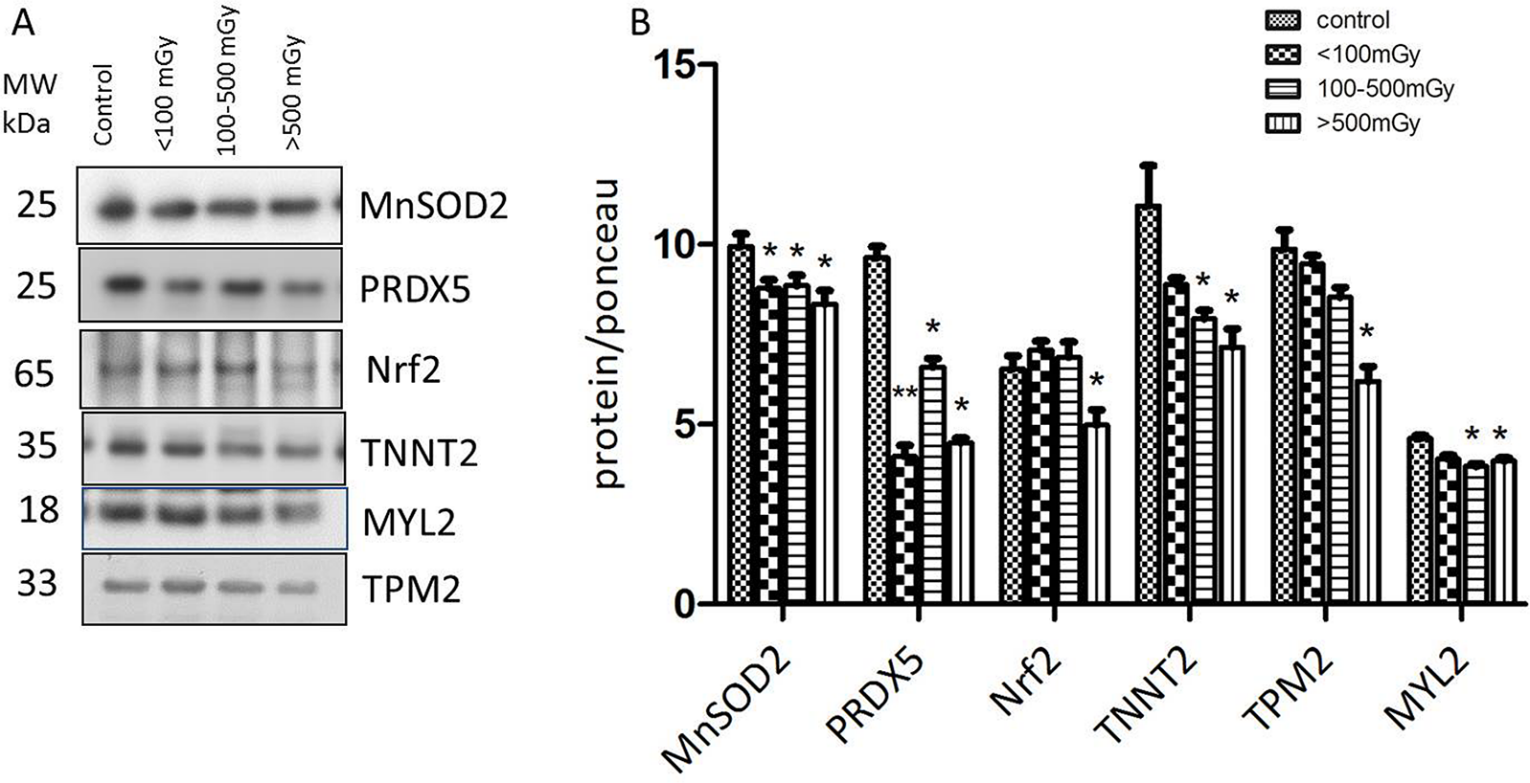
Immunoblot validation of the proteomics data The heart protein lysates from each individual sample were pooled within the dose groups and tested using anti-Troponin T (TNNT2), anti-Tropomyosin 2 (TPM2), anti- Myosin light chain (MYL2), anti-Mn superoxide dismutase (SOD2), and anti-Peroxiredoxin 5 (PRDX5) (**A**).The columns represent the average ratios of relative protein expression in control and irradiated samples. The amount of the total protein was measured by Ponceau S staining for accurate comparison between the groups. The error bars represent standard error of the mean (+ SEM) (**B**) (*t*-test; *p < 0.05, **p < 0.01; *n* = 3).

Consistent with the proteomics data, immunoblotting showed markedly decreased levels of the antioxidant defence proteins peroxiredoxin 5 (PRDX 5), and superoxide dismutase 2 (SOD2) after irradiation (Figure 3). The expression of transcription factor Nrf2, the central regulator of the antioxidative response, was significantly downregulated in the highest dose group (Figure 3). Significantly reduced expression of structural proteins myosin light chain 2 (MYL2), tropomyosin 2 (TPM2) and troponin T (TNNT2) was found in the highest dose group (Figure 3).

### Irradiation enhances protein oxidation

**Figure 4:**
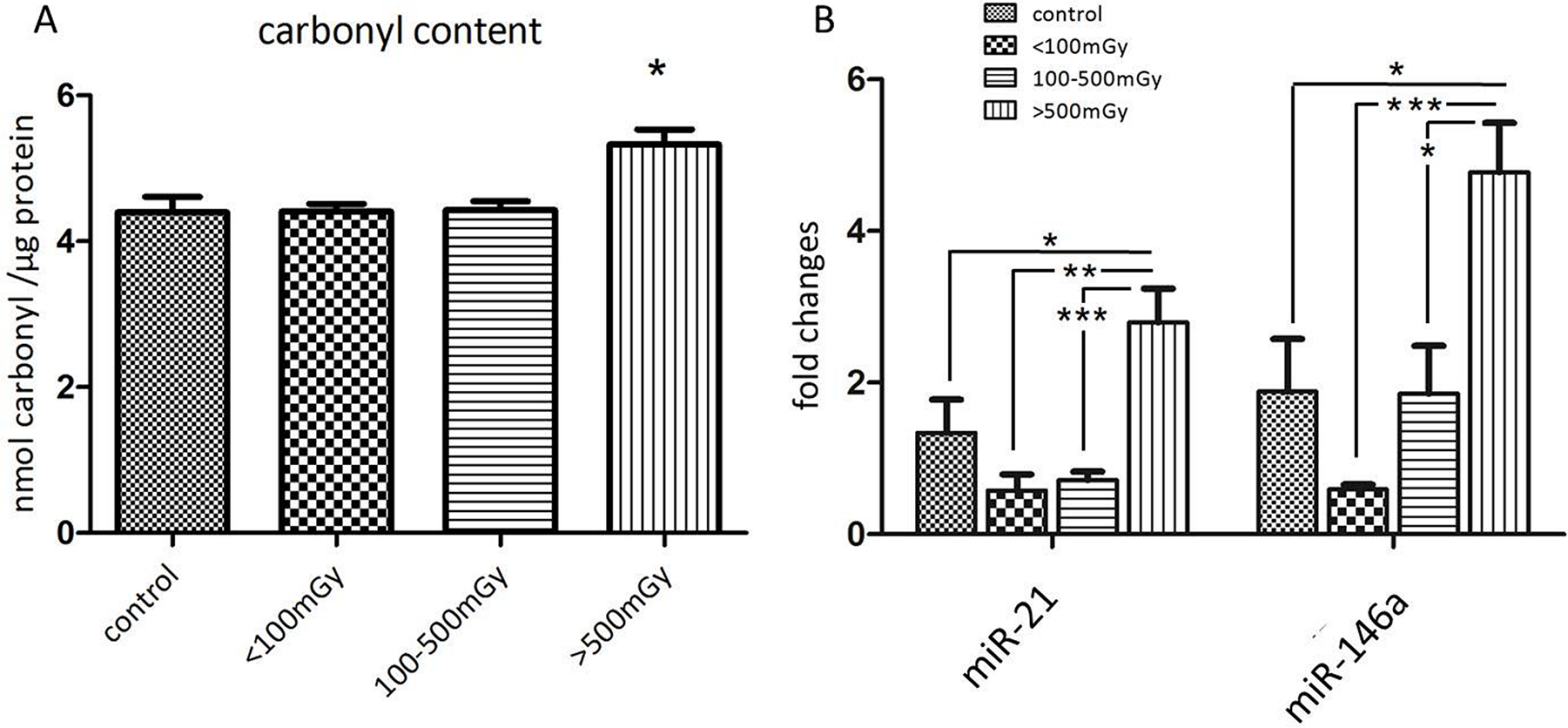
Analysis of the protein carbonyl levels and miR-21 and miR-146a in different dose groups The total amount of carbonylated protein was measured in individual samples from each dose group. The samples in the control group were run in two technical replicates. Significantly increased level of carbonylated proteins was shown in the dose group > 500 mGy (**A**). Analysis of miR-21 and miR-146a from samples of all dose groups showed significant upregulation of both miRNAs in the dose group > 500 mGy (**B**) The error bars represent standard error of the mean (+ SEM) (*t*-test; * *p* < 0.05; **p < 0.01; *** *p* < 0.001).

As the proteomics and immunoblotting data indicated alterations in the oxidative stress response, the level of protein carbonylation (protein oxidation marker) was measured in the pooled samples from each of the different dose groups. A significant increase in protein carbonylation was found in the highest dose group compared to the control (Figure 4A).

### Cardiac miRNAs are altered in irradiated hearts

MicroRNAs miR-21 and miR-146a are potential biomarkers of heart disease [17–19]. The expression of miR-21 and miR-146a was significantly upregulated in the highest dose group compared to the control and lower dose groups (Figure 4B, Supplementary Tables S8–S9).

### Transcription factor PPAR alpha is inactivated by irradiation

**Figure 5:**
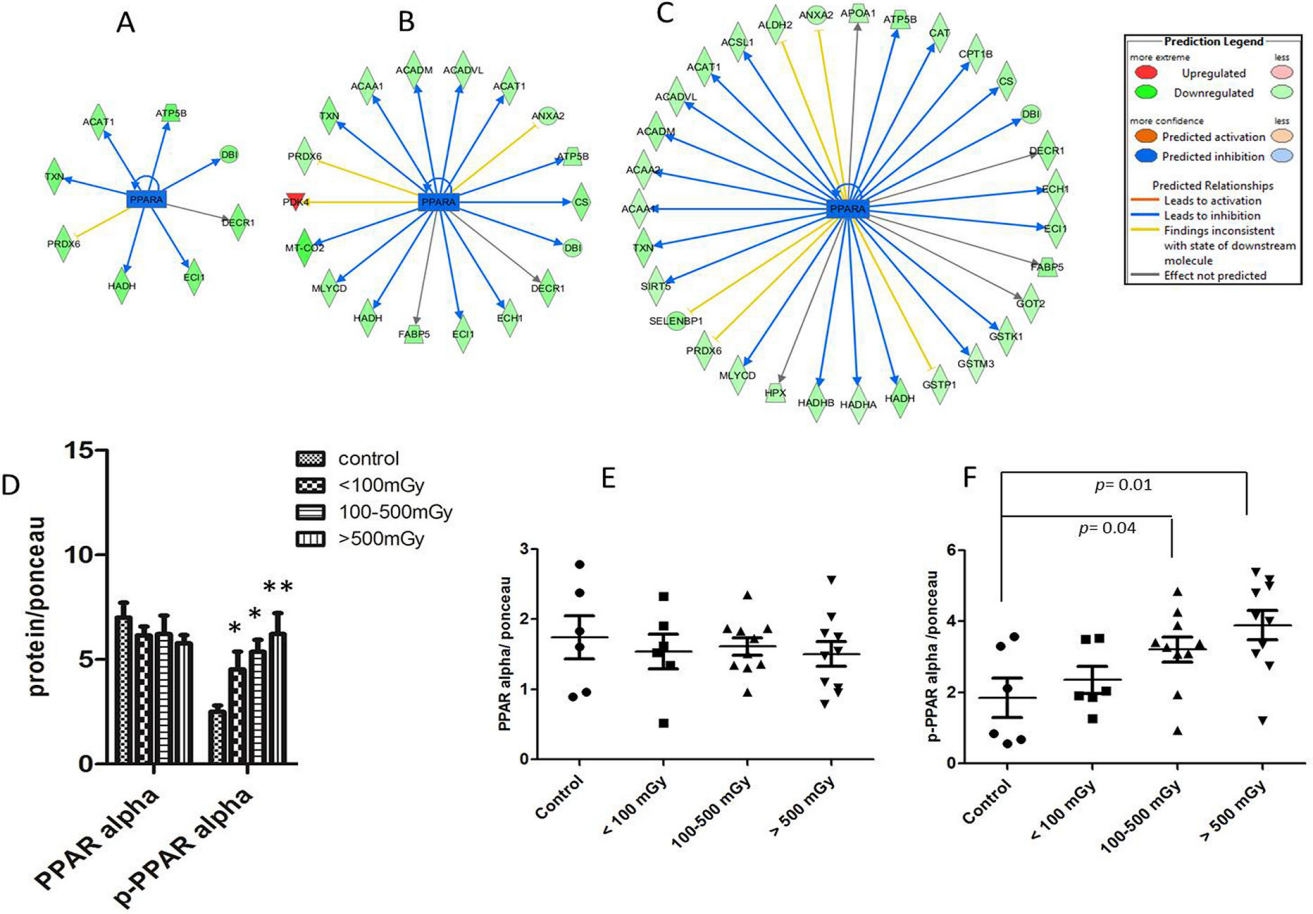
Analysis of the activation status of PPAR alpha IPA prediction of inactivation of PPAR alpha based on the deregulated proteins from proteomics analysis in the dose groups < 100 mGy (**A**), 100–500 mGy (**B**) and > 500 mGy (**C**). The upregulated proteins are marked in red and the down-regulated in green. The blue colour of the node (PPAR alpha) indicates inactivation. The list of proteins is available in Supplementary Tables S2–S4. Immunoblot analysis of total and phospho-PPAR alpha (Ser12) in pooled samples is shown (**D**). The columns represent the average ratios of relative protein expression in control and irradiated samples. Immunoblot analysis of total PPAR alpha (**E**) and phospho-PPAR alpha (**F**) in individual samples from each dose group is shown. The icons represent individual samples in different dose groups. The samples in the control group were run in two technical replicates. The amount of the total protein was confirmed by Ponceau S staining for accurate comparison between the groups (*t*-test; *p < 0.05).

Analysis of deregulated proteins predicted a significant inactivation of PPAR alpha in all exposed groups (Figure 5A–5C). The number of PPAR alpha target proteins found to have altered expression increased in a dose-dependent manner (Figure 5A–5C). Phosphorylation of PPAR alpha leads to its deactivation in the heart [20]. In agreement with the predicted inactivation, the analysis showed a significant increase in the phosphorylated form of PPAR alpha in pooled samples representing different irradiated groups (Figure 5D). To confirm this at the individual level, the expression of total PPAR alpha and its phosphorylated form were measured separately in all samples (Supplementary Figure S4). The total amount of PPAR alpha was not changed by irradiation (Figure 5E) but there was a significant increase in phospho-PPAR alpha in the irradiated samples in the highest dose groups (Figure 5F).

## DISCUSSION

The aim of the present study was to elucidate potential biological mechanisms involved in the radiation-induced IHD in human. For this purpose, we analysed post-mortem samples from the cardiac left ventricle taken from Mayak workers previously exposed to different external radiation doses. This study shows that chronic radiation exposure is able to alter the heart proteome in a dose-dependent manner. The data indicate pronounced radiation-induced changes in proteins involved in the heart function and structure, thereby supporting epidemiological evidence of a significant dose-dependent increase in the IHD incidence reported in the Mayak cohort [2, 3, 21].

In agreement with our previous data obtained in mouse models [12–14, 22, 23] the proteomics analysis shows downregulation of several mitochondrial proteins. The number of deregulated mitochondrial proteins was increased in a dose-dependent manner, indicating increasing mitochondrial dysfunction, a critical pathologic event in IHD [10]. In particular, the expression of proteins belonging to mitochondrial complexes I and III were significantly downregulated. We have previously reported that local heart irradiation in mice induces persistent functional and proteome alterations in cardiac mitochondria that are associated with reduced activity of complexes I and III [13]. Ischemic damage to the heart is also associated with defaults in the activity of complexes I and III [24, 25]. As the control group used in this study was also suffering from IHD, it can be suggested that chronic radiation worsens the respiratory complex impairment, at least on the proteome level.

A decrease in the mitochondrial respiration rate has been reported to enhance reactive oxygen species (ROS) production [26]. We have shown previously that local heart irradiation permanently increases mitochondrial ROS levels in mice [12, 13]. This study now shows a dose-dependent decrease in the level of the antioxidant response regulator protein Nrf2. The expression of many Nrf2 target proteins, as well as other proteins of oxidative response, were significantly downregulated (up to 50%) in the highest dose group. These included superoxide dismutases (SOD1 and SOD2), peroxiredoxins (1, 2, 3, 5, and 6), glutathione-S-transferases (kappa1, mu2, mu3, omega 1, pi1) and catalase. This marked downregulation of antioxidant capacity, coupled with increased carbonylation, suggests an increased radiation-induced ROS production. The expression of miR-146a is increased by oxidative stress [27]. This miRNA was markedly increased in the highest dose group. A target protein of miR-146a is SOD2 [27] which was indeed downregulated in this group.

Mitochondria are not only the main source and but also a target of oxidative damage. As oxidative stress-induced protein modifications such as carbonylation lead to increased protein degradation [28] or inactivation [29], increased oxidation of mitochondrial proteins may explain the vigorous downregulation in their expression seen in this study.

Mitochondria are physically associated with myofibrils, and increased mitochondrial ROS production may lead to impaired contractility through disruption of actin-myosin interactions [30]. Cytoskeletal proteins are considered to be sensitive to redox alterations and the association between oxidative stress and structural damage has been well documented [31]. The oxidation of actin has been shown to result in a strong inhibition of protein polymerisation and in complete disruption of actin-filament organisation [31]. This study shows a significant downregulation of actin isoforms together with many other structural proteins (tubulin, troponin, tropomyosin, desmin and different isoforms of light and heavy myosin). These proteins are major constituents of the contractile apparatus and the severe downregulation seen in the high-dose groups may negatively influence cardiac contractility. Cardiac troponin, a sensitive and specific marker for heart damage, is significantly reduced in the two highest dose groups, in comparison to the controls [32].

Prompted by the bioinformatics prediction based mainly on downregulated metabolic and oxidative response proteins, we show here that ionising radiation increases the level of phosphorylation of PPAR alpha and thereby inhibits this transcription factor in a dose-dependent manner [6, 20]. This is in agreement with our previous study showing that high-dose local heart irradiation impairs the cardiac fatty acid oxidation due to inhibition of PPAR alpha activity in mice [14]. The PPAR alpha pathway has been shown to influence antioxidant response and myofibrillar structure [33]. Increased oxidative stress and reduced contractility due to oxidation of myosin have been reported in PPAR alpha knock-out mice [34]. We suggest that inactivation of PPAR alpha following radiation exposure adds to the oxidative stress and structural impairment phenotype observed in irradiated hearts.

The level of PPAR alpha and that of several mitochondrial proteins is regulated by mir-21 in different cells and tissues [35–37]. We found significant upregulation in the expression of miR-21 in the left ventricle samples of the highest dose group. This corresponds to the observed downregulation of mitochondrial proteins. Increased levels of mir-21 in heart failure and ischemia have been reported [19, 38, 39].

## MATERIALS AND METHODS

### Samples

Biological samples were collected post-mortem from donors who had previously given informed consent to participate in the study and who had consented to the processing of their personal data in accordance with the Russian Federal Laws No 323-FL of 27.09.2013 and No 261-FL of 25.07.2011. The study was approved by the Southern Urals Biophysics Institute´s Institutional Review Board.

The individuals were male Mayak plutonium enrichment plant workers who were exposed only to external gamma rays. The control subjects were non-Mayak workers living in the same area. All participants were diagnosed multiple times with IHD during their lifetime and the primary cause of death was IHD. Workers exposed to internal plutonium (Pu alpha-activity in urine > 0.5 kBq), or who had been diagnosed with cancer or other major somatic diseases were excluded from the study.

All individuals were placed in a cold-room (+4°C) immediately after the death (approximately within 1 h). All autopsies were performed within first 12–24 h after the death. The cardiac left ventricle was collected at autopsy and immediately frozen. Heart tissues from 29 individuals were allocated between four dose groups as follows: 3 individuals to the control group (0 Gy), 6 to the dose group < 100 mGy, 10 individuals to the group receiving doses between 100–500 mGy, and 10 individuals to the dose group > 500 mGy (Supplementary Table S10). The smoking status and index, alcohol consumption and body mass index of each individual is indicated in Supplementary Table S10.

To expand the number of participants, formalin-fixed paraffin-embedded (FFPE) samples from 15 donors were used for miRNA analysis as follows: 3 individuals from control group, 3 individuals from the dose group <100 mGy, 4 individuals from the group representing doses between 100–500 mGy, and 5 individuals from the dose group > 500 mGy (Supplementary Table S10). Four participants (5, 16, 26 and 27) were donors of both frozen and FFPE samples.

### Protein extraction

Frozen heart samples were lysed as described previously [14]. Cardiac left ventricle was ground to a fine powder with a cold (−20°C) mortar and pestle before being suspended in lysis buffer (SERVA) [14]. Protein concentration was determined by the Bradford assay following the manufacturer's instructions (Thermo Fisher).

### Protein purification and mass spectrometry

Protein lysates (10 μg) were digested using a modified filter-aided sample preparation (FASP) protocol [40]. Briefly, the samples were reduced with 10 mM DTT at 60°C for 30 min, followed by alkylation with 15 mM iodoacetamide for 30 min at room temperature [40]. Samples were diluted using 8 M urea in 0.1 M Tris/HCl, pH 8.5, and centrifuged using a 30 kDa cut-off filter (Pall Corporation). After washing with 8 M urea in 0.1 M Tris/HCl, pH 8.5, and with 50 mM ammonium bicarbonate (ABC), the proteins were initially digested on the filter with 1 μg Lys-C (Wako Chemicals GmbH) in 50 mM ABC at room temperature, followed by addition of 2 μg trypsin (Promega) and digestion overnight at 37°C. Tryptic peptides were collected by centrifugation and acidified with trifluoric acid (TFA) to a pH of 2.0. Samples were stored at −20°C.

Prior to LC-MS/MS analysis the samples were centrifuged (16,000 g) for 5 min at 4°C. Each sample (0.5 μg) representing one donor was analysed separately on a LTQ OrbitrapXL (Thermo Fisher Scientific) coupled to Ultimate 3000 nano-HPLC (Dionex) as described previously [41].

### Label-free quantification

The raw files of the individual measurements were loaded to the Progenesis QI software and analysed as described previously [42, 43]. Briefly, peptide features in the individual runs were aligned in order to reach a maximum overlay of at least 85%. After feature detection, the singly charged features and features with charges higher than +7 were excluded. The samples were grouped according to the radiation dose as described above. Protein identification was performed using the Mascot search engine (Matrix Science, version 2.5.0) with the Ensembl Human database (version 68, 40047886 residues, 105288 sequences).

The following search parameters were used: 10 ppm peptide mass tolerance and 0.6 Da fragment mass tolerance, one missed cleavage was allowed, carbamidomethylation (C) was set as fixed modification, and oxidation (M) and deamidation (N, Q) were allowed as variable modifications. Search results were reimported into the Progenesis QI software and the resulting summed normalised abundances of unique peptides for every single protein were used for the calculation of abundance ratios and statistical analysis (Student's *t*-test).

A principal component analysis (PCA) was performed using Progenesis QI software (http://www.nonlinear.com), based on all features with charges +2 to +7 resulting in the PC1 of 23.65% and PC2 of 8.36%.

For final quantifications, proteins with ratios greater than 1.30-fold or less than 0.77-fold (*t*-test; *p* < 0.05) were defined as being significantly differentially expressed. The FDR (q value) calculation was used to adjust *p*-values [44, 45]. The calculation was performed using modified BenjaminiHochberg created by Manuel Weinkauf (https://marum.de/Software_and_Programs.html), licensed under a Creative Commons Attribution-NonCommercial-ShareAlike 3.0 Unported License (http://creativecommons.org/licenses/by-nc-sa/3.0/deed.en_GB). All *p*-values below the corrected significance level q were considered to represent significant results.

### Protein-protein interaction and signalling network

For deregulated proteins, protein-protein interaction and signalling networks were analysed by the software tool INGENUITY Pathway Analysis (IPA) (http://www.INGENUITY.com) [46] and the search tool STRING version 10 (http://string-db.org), coupled to the Reactome database (http://www.reactome.org) [47].

### Immunoblot analysis

Protein lysates from pooled or individual (total and phospho-PPAR alpha) samples were analysed by immunoblotting. For pooled samples, similar amount of protein from each individual belonging to the same radiation dose group (control, < 100 mGy, 100–500 mGy and > 500 mGy) was combined into a batch representing that group. Proteins separated by 4–12% SDS-PAGE were transferred to nitrocellulose membranes (GE Healthcare) using a TE 77 semidry blotting system (GE Healthcare) at 1 mA/cm for 1h. The membranes were blocked using 3 % BSA in TBS, pH 7.4, for 1 h at room temperature, washed three times in 10 mM Tris-HCl, pH 7.4, 150 mM NaCl for 5 min and incubated overnight at 4°C with primary antibodies using dilutions recommended by the manufacturer (Abcam). Immunoblot analysis of heart protein lysate was performed using anti-PPAR alpha (# ab2779), anti-phospho-PPAR alpha (Ser12)(# ab3484), anti-troponin T(# ab156852), anti-SOD2 (# ab13533), anti-peroxiredoxin 5 (# ab119712), anti-myosin light chain 2 (# ab 92721), anti-tropomyosin 2 (# ab96073) and anti-Nrf2 (# ab31163). After washing three times, the blots were incubated with the appropriate horseradish peroxidase-conjugated or alkaline phosphatase-conjugated anti-mouse, anti-rabbit or anti-goat secondary antibody (Santa Cruz Biotechnology) for 2 h at room temperature and developed using the ECL system (GE Healthcare) or 1-step^TM^ NBT/BCIP method (ThermoFisher) following standard procedures. Reversible Ponceau staining was used as the loading control. Quantification of digitised images of immunoblot bands was done using ImageJ (http://rsbweb.nih.gov/ij/). Three technical replicates were performed from each pooled sample. For individual analysis, the samples in the control group were run in two technical replicates, all others in one technical replicate.

### Protein carbonylation analysis

To detect the level of protein oxidation, protein carbonylation was measured using the assay kit (Biovision) according to the manufacturer´s instructions.

### RNA isolation from FFPE blocks and TaqMan-miRNA assays

Heart tissue was immediately fixed in 4% buffered formalin for 24 h and dehydrated with a graded series of ethanol before embedding in paraffin. FFPE blocks were stored in the dark at room temperature. For miRNA analysis, multiple 10 μm sections were cut after initial trimming to remove air exposed surfaces. Total RNA was isolated using phenol chloroform gradient as described previously [48] and quantified using NanoDrop spectrophotometer (PeqLab Germany).

Quantitative PCR (Applied Biosystems, Forster City, CA, USA) was performed to analyse the expression of miR-21 (# 4427975, assay id 000397) and miR-146a (#4427975, assay id 000468) with the StepOnePlus Detection System (Applied Biosystems, Foster City, CA) according to the manufacturer´s instructions. Relative expression values of each miRNA were calculated using the 2–ΔΔCT method, normalised to the control miRNA RNU44 (# 4427975, assay id 001094) as described earlier [49]. Relative expression values from control and exposed groups were used for further calculations. All samples were analysed at least in duplicate.

### Statistical analysis

Comparative analysis of the data was carried out using the Student´s *t*-test (two-paired and unpaired). The significance levels were *p < 0.05 (5%); **p < 0.01 (1%) and ***p < 0.001 (0.1%). The error bars represent the standard error of the mean (± SEM).

### Data availability

The raw MS data can be accessed from the RBstore database http://www.storedb.org/store_v3/study.jsp?studyId=1038

## CONCLUSIONS

This data emphasizes the critical role of PPAR alpha and defect fatty acid oxidation in the radiation-induced IHD. Furthermore, the reduced energy flow from beta oxidation may not be compensated with increased uptake of glucose as the majority of the enzymes in the glycolysis pathway were also downregulated in a dose-dependent manner. This may lead to severe ATP depletion in the irradiated heart. Improving the function of PPAR alpha may serve as a useful preventive tool in radiation-induced IHD.

## SUPPLEMENTARY MATERIALS FIGURES AND TABLES




