# Supplementary material for: A dose-dependent perturbation in cardiac energy metabolism is linked to radiation-induced ischemic heart disease in Mayak nuclear workers
Source: Oncotarget. 2016 Jul 6;8(6):9067–78. doi: 10.18632/oncotarget.10424 (PMC5354715; doi:10.18632/oncotarget.10424)
Supplement: Supplementary file 1 [file oncotarget-08-9067-s001.pdf]

A dose-dependent perturbation in cardiac energy metabolism is linked to radiation-induced ischemic heart disease in Mayak nuclear workers

Supplementary Materials

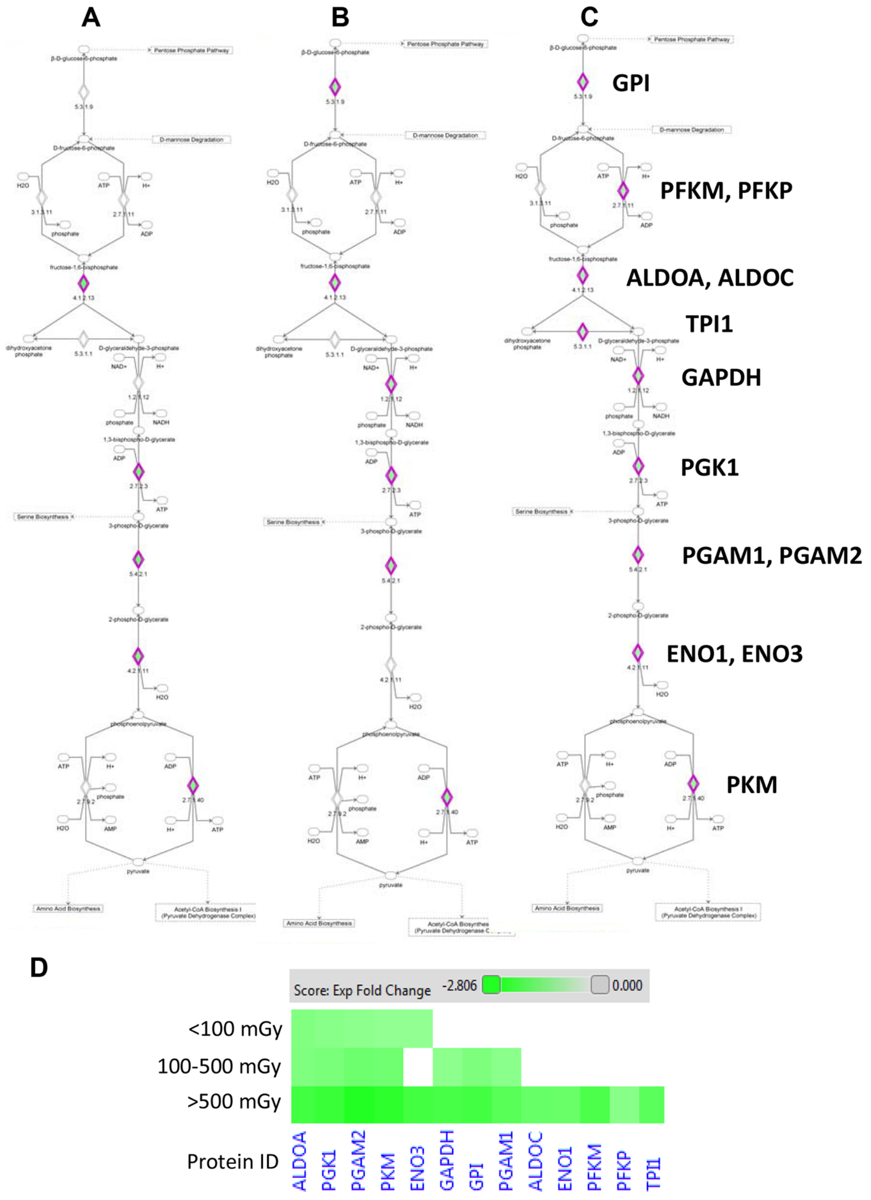

Supplementary Figure S1: Pathway analysis of significantly differentially expressed proteins involved in glycolysis. A dose-dependent increase in the number of deregulated glycolytic proteins is observed. The downregulated proteins (marked in green) are indicated with diamonds at < 100 mGy (**A**) 100–500 mGy (**B**) and > 500 mGy (**C**). The downregulated proteins glucose-6-phosphate isomerase (5.3.1.4; GPI), 6- phosphofructokinase 1 (2.7.1.11; PFKM, PFKP), fructose-bisphosphate aldolase, class I (4.1.2.13; ALDOA, ALDOC), triosephosphate isomerase (5.3.1.1; TPI1), glyceraldehyde 3-phosphate dehydrogenase (1.2.1.12; GAPDH), phosphoglycerate kinase (2.7.2.3; PGK1), 2,3-bisphosphoglycerate-dependent phosphoglycerate mutase (5.4.2.1; PGAM1, PGAM2), enolase (4.2.1.11; ENO1, ENO3), and pyruvate kinase (2.7.1.40; PKM) are indicated in the map with the corresponding Protein ID (bold). The heat map (**D**) shows the downregulated proteins in each dose group. (http://www.INGENUITY.com). The protein fold changes are displayed using a green colour gradient, where darker green corresponds to higher fold changes compared to control. White boxes represent not significantly deregulated proteins.

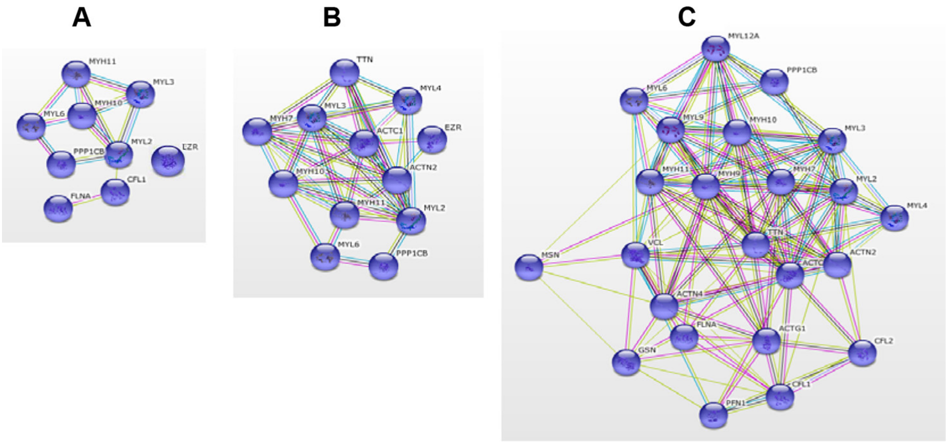

Supplementary Figure S2: Protein-protein interaction analysis of the significantly differentially expressed structural proteins. The deregulated mitochondrial proteins, indicated with blue circles, are shown with their networks at < 100 mGy (**A**), 100–500 mGy (**B**) and > 500 mGy (**C**). Association networks were analysed by the STRING software tool (http://string-db.org).

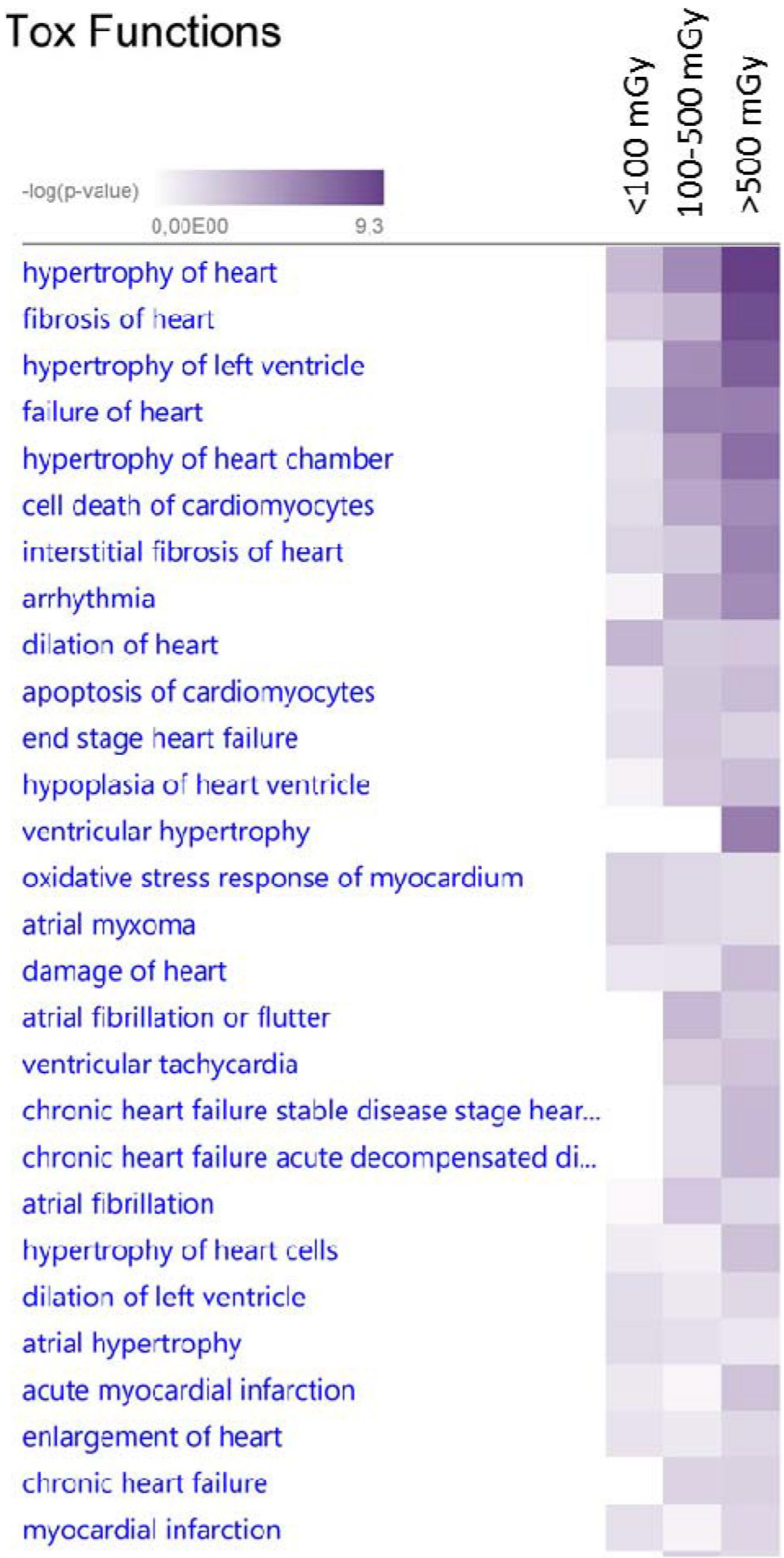

Supplementary Figure S3: Ingenuity pathway analysis of cardiac toxicity analysis of the significantly differentially expressed structural proteins. Among the altered proteins categorised in different cardiotoxicity groups, those involved in cardiac hypertrophy, cardiac fibrosis and cell death represent the majority.

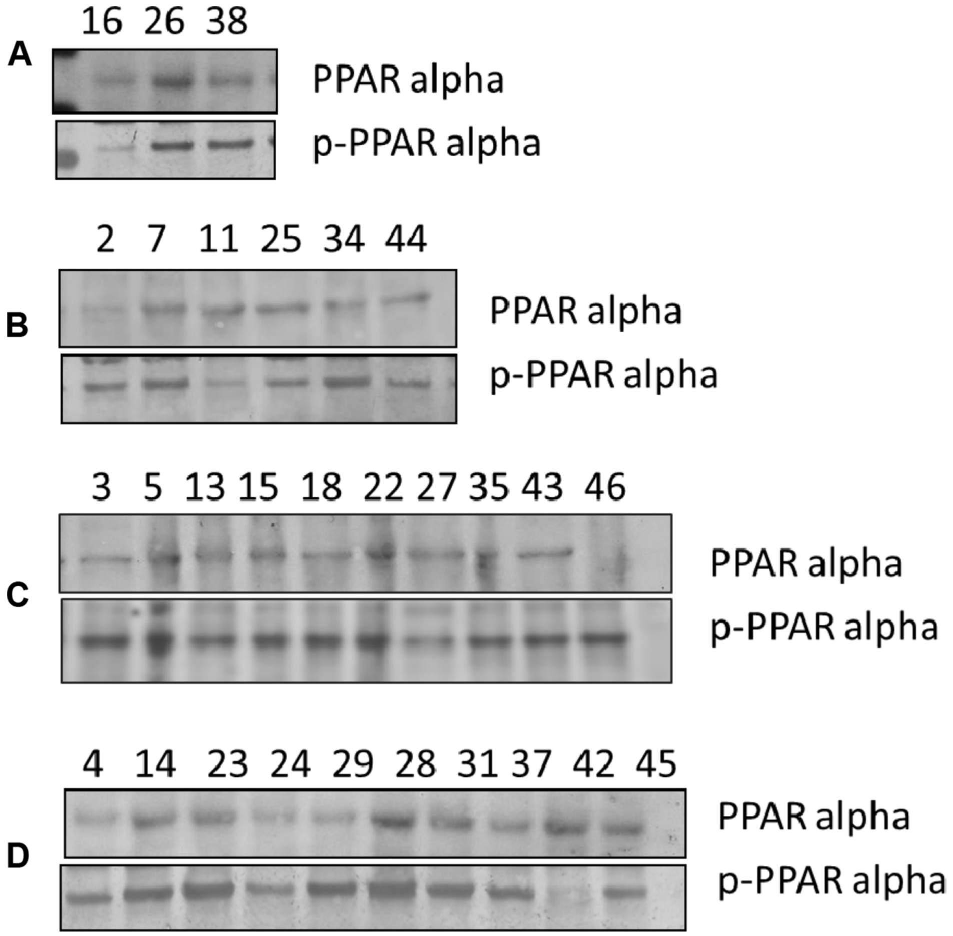

Supplementary Figure S4: Immunoblotting analysis of total and phospho-PPAR alpha levels. The heart protein lysates from individual samples from control group (**A**) group exposed to < 100 mGy (**B**), group exposed to 100–500 mGy (**C**) and group exposed to > 500 mGy (**D**) were tested using anti-PPAR alpha and anti-phospho-PPAR alpha (Ser-12) as described in Materials and Methods.

Supplementary Table S1: All identifed and quantified proteins. The proteins identified by at least 2 unique peptides are indicated in bold. See Supplementary_Table_S1

Supplementary Table S2: Significantly differently expressed proteins quantified by label-free approach after < 100 mGy exposure. The proteins listed in the table were identified and quantified by two or more unique peptides; *t*-test *p* value is shown. All *p*-values below the corrected significance level *q** considered as significant results. See Supplementary_Table_S2

Supplementary Table S3: Significantly differently expressed proteins quantified by label-free approach after 100–500 mGy exposure. The proteins listed in the table were identified and quantified by two or more unique peptides; *t*-test *p* value is shown. All *p*-values below the corrected significance level *q** considered as significant results. See Supplementary_Table_S3

Supplementary Table S4: Significantly differently expressed proteins quantified by label-free approach after > 500 mGy exposure. The proteins listed in the table were identified and quantified by two or more unique peptides; *t*-test *p* value is shown. All *p*-values below the corrected significance level *q** considered as significant results. See Supplementary_Table_S4

Supplementary Table S5: Comparison of top networks of all exposed groups (IPA). The score is a numerical value used to rank networks according to their degree of relevance to the network eligible molecules in proteome dataset. See Supplementary_Table_S5

Supplementary Table S6: Comparison of canonical pathways in exposed groups (IPA). The ratio refers to the number of proteins from the list that maps to the pathway divided by the total number of proteins that map to the same pathway. The pathways are ranked by the highest total significancies across the groups. The *p*-value is calculated using Fisher's exact test. See Supplementary_Table_S6

Supplementary Table S7: Comparison of cardiac toxicity function of all exposed groups (IPA). The *p*-value is calculated by Fisher's exact test. See Supplementary_Table_S7

Supplementary Table S8: miR-21 analysis of FFPE samples of individuals from the control and irradiated groups. The bold numbers represent the shared donors whose samples used in both miRNA analysis and proteomics. See Supplementary_Table_S8

Supplementary Table S9: miR-146a analysis of FFPE samples from individuals in the control and irradiated groups. The bold numbers represent the shared donors whose samples used in both miRNA analysis and proteomics. See Supplementary_Table_S9

Supplementary Table S10: Detailed information of the donors for frozen tissue samples (A) and for FFPE tissue samples (B). Samples from the donors 5, 16, 26 and 27 indicated in bold were used both for proteomics (frozen) and miRNA analysis (FFPE). Smoking index was calculated as the mean number of cigarette packs smoked in a day times years of smoking. The smoking index was measured by pack-years and for 'never-smokers' was equated with zero. BMI is a measure used for assessing underweight, normal weight or overweight of an individual. BMI for a person is defined as body mass in kilograms divided by the square of their height in meters. The 'normal BMI' is in the range 18.5–24.99 kg/m^2. BMI was taken as of the date of death (or the 5 years prior to the date of death). BMI was as follows: 0 for unknown, 1 for underweight (< 18.5 kg/m^2); 2 normal weight (18.5–25 kg/m^2); 3 for overweight (25–30kg/m^2); 4 for more overweight (> 30 kg/m^2). See Supplementary_Table_S10
